# Effect of an Interscalene Block Combined With Intravenous Dexamethasone on Pain After Rotator Cuff Repair

**DOI:** 10.7759/cureus.79265

**Published:** 2025-02-19

**Authors:** Kentaro Ohuchi, Tatsuru Tomioka, Junichi Inoue, Kunio Ebata, Naohisa Miyakoshi

**Affiliations:** 1 Department of Orthopedic Surgery, Yokote Municipal Hospital, Yokote, JPN; 2 Department of Orthopedic Surgery, Akita University Graduate School of Medicine, Akita, JPN

**Keywords:** arthroscopic rotator cuff repair, dexamethasone, interscalene block, intravenous injection, postoperative pain

## Abstract

Background

Shoulder joint surgery is associated with severe postoperative pain, necessitating effective pain control methods. While interscalene block is a widely used technique for postoperative pain relief, its duration is limited, and rebound pain often occurs. Combining interscalene block with dexamethasone has shown promise in extending pain relief, but the relative effectiveness of intravenous versus mixed administration remains unclear. This study aimed to evaluate the effects of intravenous dexamethasone combined with interscalene block on pain control after arthroscopic rotator cuff repair (ARCR).

Methodology

A retrospective study was conducted on 73 ARCR patients who received general anesthesia and interscalene block between 2018 and 2023. Patients were divided into two groups: those receiving an interscalene block with intravenous dexamethasone (group D, *n *= 41) and those receiving an interscalene block alone (group C, *n *= 32). Primary outcomes included postoperative rescue drug use and the time to first rescue drug use. Secondary outcomes included the frequency of rescue drug use within 12, 24, and 72 hours post-surgery. Statistical analysis was performed using the Mann-Whitney U test and the chi-square test.

Results

Group D had a significantly lower rate of rescue drug use (26, 63%) compared to group C (31, 97%; *P *< 0.001). The time to first rescue drug use was significantly longer in group D (21.7 ± 9.8 hours) than in group C (9.3 ± 8.3 hours, *P *< 0.001). The frequency of rescue drug use within 12, 24, and 72 hours post-surgery was also significantly lower in group D (*P *< 0.001). No complications were observed in either group.

Conclusions

Intravenous dexamethasone combined with interscalene block significantly reduced the need for postoperative painkillers and prolonged the duration of pain relief after ARCR. This combination is a promising method for effective postoperative pain management.

## Introduction

Shoulder joint surgery is known to cause severe postoperative pain, and pain control is important [1]. It has also been reported that postoperative pain from a rotator cuff tear is stronger than the pain following lower limb arthroplasty [2]. Methods reported to reduce postoperative pain include the use of opioids, cocktail injections, and peripheral nerve blocks [3]. Ultrasound-guided interscalene block is a widely used method to achieve good postoperative pain relief [4]. However, it has been pointed out that the effect is short when used once, and rebound pain can occur [5]. Although an interscalene catheter can be placed to provide sustained pain relief, there are concerns about side effects associated with catheter placement [6].

In recent years, there have been several reports showing that the interscalene block effect can be extended by combining it with dexamethasone [7-9]. While methods exist to add dexamethasone to anesthetic drugs or administer it intravenously, the relative effectiveness and associated side effects remain undefined. The purpose of this study was to investigate the effect of interscalene block combined with intravenous dexamethasone injection on pain after arthroscopic rotator cuff repair.

## Materials and methods

Approval for this study was granted by the institutional review board of our hospital (approval number 2302), and all subjects gave their informed consent to participate. Between 2018 and 2023, 73 cases of rotator cuff tears treated with arthroscopic rotator cuff repair under general anesthesia, with a preoperative interscalene block, were included in the study. Cases in which cocktail injections were administered simultaneously, cases of reoperation, and cases with diabetes were excluded. General anesthesia was administered by several anesthesiologists, and the interscalene block was performed by either the anesthesiologist or the surgeon. All surgeries were performed by a single shoulder surgeon.

The anesthetic used for the interscalene block was either 10 mL of ropivacaine or 10 mL of levobupivacaine mixed with 10 mL of saline, depending on the anesthesiologist's preference. The subjects were divided into two groups: 41 patients in the group receiving interscalene block plus dexamethasone 3.3 mg (group D), and 32 patients in the group receiving interscalene block alone (group C). Dexamethasone was administered intravenously as a single bolus dose just before the interscalene block. No additional doses were given postoperatively. 

All surgeries were performed in the beach chair position. Depending on the size of the rotator cuff tear, arthroscopic rotator cuff repair was performed using one of the three techniques.

Single-row technique

Anchors are placed in a single row along the footprint of the rotator cuff, with sutures passing through the tendon to secure it to the bone.

Suture bridge technique

Additional lateral anchors are used to create a broader tendon-to-bone contact area, enhancing biomechanical stability.

Triple-row technique

A combination of medial, central, and lateral row anchors is used to optimize tendon fixation and footprint coverage, aiming for improved healing. From the day after surgery, acetaminophen and pregabalin were regularly administered orally. For pain relief, rescue drugs were provided based on patient preference, which included oral loxoprofen, diclofenac suppositories, or intramuscular ketoprofen injections.

The primary outcome was a comparison between the two groups regarding the use of rescue drugs for postoperative pain. Secondary outcomes included the time to first use of rescue drugs, the number of rescue drug uses within 12, 24, and 72 hours post-surgery, as well as intraoperative and postoperative complications.

Statistical analysis was performed using R Commander (R Foundation for Statistical Computing, Vienna, Austria) [10]. The Mann-Whitney U test was used to compare age, body mass index (BMI), Japanese Orthopedic Association (JOA) pain score, Shoulder 36 pain score, operation time, time to first use of rescue drugs, and the number of rescue drug uses. The JOA pain score assesses subjective pain levels reported by the patient during daily activities or at rest, ranging from 0 to 30 points, with higher scores indicating less pain and better shoulder function. The Shoulder 36 pain score reflects the extent to which pain interferes with the patient's physical and social functions, with higher scores indicating less pain and better overall shoulder function. In addition, the chi-square test was used to compare gender, use of rescue drugs, type of anesthetic used for the interscalene block, and rotator cuff tear size. Statistical significance was set at *P *< 0.05.

## Results

**Figure 1:**
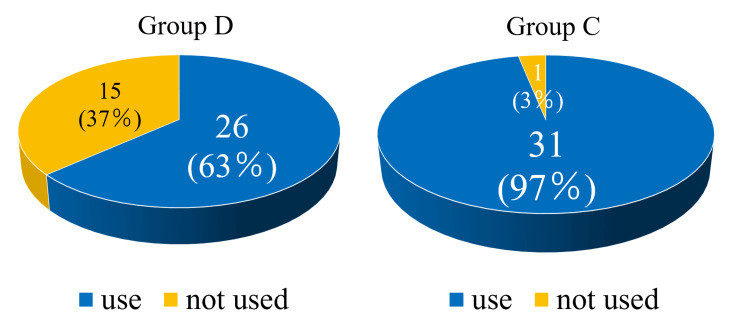
The rate of postoperative rescue drug use. The rate of postoperative rescue drug use was significantly lower in group D (63%) compared to Group C (97%) (*P* < 0.001). Group D: Patients receiving an interscalene block with intravenous dexamethasone. Group C: Patients receiving an interscalene block alone. Statistical analysis was performed using the chi-square test.

**Figure 2:**
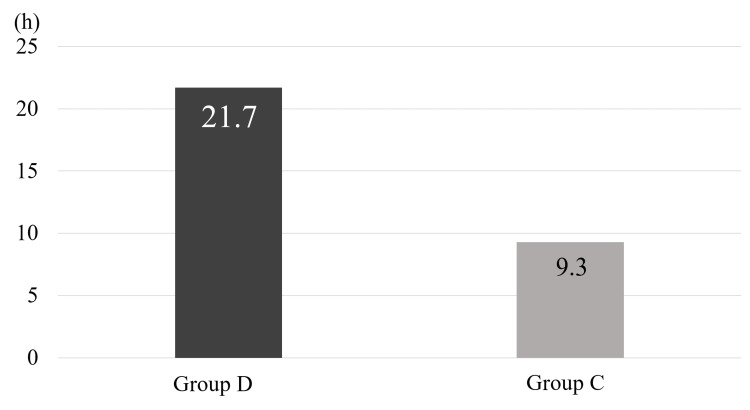
The time to first use of rescue drugs. The time to first use of rescue drugs was significantly longer in group D (21.7 ± 9.8 hours) compared to group C (9.3 ± 8.3 hours) (*P* < 0.001). Group D: Patients receiving an interscalene block with intravenous dexamethasone. Group C: Patients receiving an interscalene block alone. Statistical analysis was performed using the Mann-Whitney U test.

**Figure 3:**
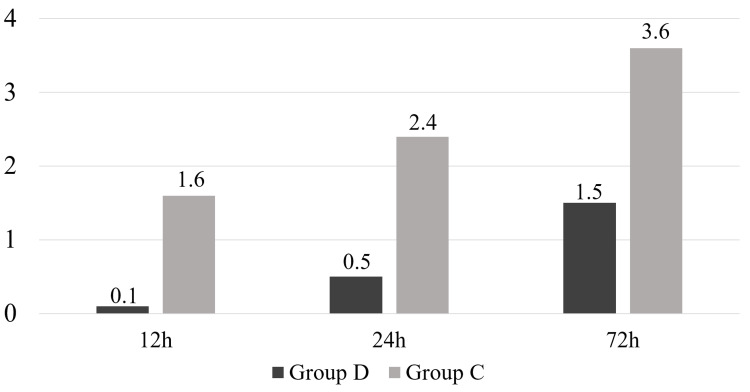
The frequency of rescue drug use. The frequency of rescue drug use at 12, 24, and 72 hours post-surgery was significantly lower in group D (0.1 ± 0.4, 0.5 ± 0.9, and 1.5 ± 1.8 times, respectively) compared to group C (1.6 ± 1.4, 2.4 ± 1.8, and 3.6 ± 2.3 times, respectively) (*P* < 0.001). Group D: Patients receiving an interscalene block with intravenous dexamethasone. Group C: Patients receiving an interscalene block alone. Statistical analysis was performed using the Mann-Whitney U test.

There were no significant differences between the two groups in age, sex, BMI, preoperative pain score, rotator cuff tear size, type of anesthetic used, or operation time (Table 1). The rate of postoperative rescue drug use was 63% in group D and 97% in group C, with the rate being significantly lower in group D (*P *< 0.001) (Figure 1). The time to first use of rescue drugs was 21.7 ± 9.8 hours in group D and 9.3 ± 8.3 hours in group C, which was significantly longer in group D (*P *< 0.001) (Figure 2). The frequency of rescue drug use within 12, 24, and 72 hours after surgery was significantly lower in group D (0.1 ± 0.4, 0.5 ± 0.9, and 1.5 ± 1.8 times, respectively) compared to group C (1.6 ± 1.4, 2.4 ± 1.8, and 3.6 ± 2.3 times, respectively) (*P *< 0.001) (Figure 3). No complications from the interscalene block occurred in either group.

**Table 1:** Patients’ demographic variables. There were no significant differences in demographic variables between the two groups. Group D consisted of patients receiving an interscalene block with intravenous dexamethasone, while Group C received an interscalene block alone. Values are presented as mean ± standard deviation or as the number of patients. Statistical analysis was performed using the Mann-Whitney U test and the chi-square test. JOA, Japanese Orthopedic Association

	Group D (*n* = 41)	Group C (*n* = 32)	*P*-value
Age (years)	65.9 ± 10.2	64.3 ± 7.7	0.169
Sex (male/female)	33/8	24/8	0.574
Body mass index (kg/m^2^)	23.9 ± 3.3	25.5 ± 4.2	0.074
JOA pain score	9.6 ± 4.1	9.1 ± 3.2	0.519
Shoulder 36 pain score	2.8 ± 0.9	2.7 ± 1.1	0.693
Size of tear (small, medium/ large, massive)	32/9	27/5	0.422
Type of anesthetic used (ropivacaine/levobupivacaine)	23/18	17/15	0.8
Operation time (minutes)	133.3 ± 49.4	126.4 ± 39.6	0.508

## Discussion

Postoperative pain management remains a critical challenge in shoulder surgery, particularly after arthroscopic rotator cuff repair. Interscalene block is widely used as an effective pain control technique, but its duration is often limited, leading to rebound pain when the effect wears off. Various adjuncts, including opioids and steroid injections, have been investigated to enhance its analgesic duration. Among these, dexamethasone has gained attention for its potential to prolong nerve block effects and reduce postoperative pain. The results of this study demonstrated that the analgesic effect of interscalene block combined with intravenous dexamethasone prolonged the block's pain relief effect and significantly reduced the rate and frequency of rescue drug use after arthroscopic rotator cuff repair. Regarding the pharmacological mechanism of action of the addition of steroids, it has been reported that they delay the absorption of local anesthetics by promoting vascular constriction around nerves and suppressing the production of inflammatory proteins [11,12]. However, the pharmacological mechanism by which intravenous dexamethasone prolongs the analgesic effect of interscalene block remains incompletely understood.

There have been several reports examining whether the duration of the block effect differs between the addition of steroids and intravenous administration. Desmet et al. divided shoulder arthroscopic surgery patients who underwent interscalene block into three groups: an interscalene block group, a group in which dexamethasone 10 mg was added, and a group in which dexamethasone 10 mg was administered intravenously, and compared the duration of block anesthesia. They reported that the duration of anesthesia was significantly longer in the two groups in which dexamethasone was used compared to the group in which dexamethasone was not used, but there was no significant difference between the group in which dexamethasone was added and the group in which dexamethasone was administered intravenously [7]. In addition, Chun et al. compared the duration of anesthesia in shoulder arthroscopic surgery patients who underwent interscalene block between a group that received dexamethasone 5 mg and a group that received intravenous dexamethasone 5 mg and reported that the duration of anesthesia was significantly longer in the group that received dexamethasone [13]. This study compared a group that received intravenous dexamethasone with a group that did not, and it is not possible to say whether this is superior or inferior in effectiveness compared to mixing dexamethasone with an anesthetic drug; however, it may at least be possible to expect an extended effect of block anesthesia compared to when dexamethasone is not used.

There is no consensus on the effect of different doses of dexamethasone on the duration of block anesthesia. Desmet et al. compared the use of an interscalene block in combination with an intravenous dexamethasone injection in arthroscopic shoulder surgery, dividing patients into four groups: a non-use group and groups receiving 1.25, 2.5, or 10 mg of dexamethasone. As a result, no significant difference in the duration of anesthesia was observed between the group that received 1.25 mg of dexamethasone and the non-use group. However, the group that received 2.5 mg or more of dexamethasone reported a significantly longer duration of anesthesia [14]. In addition, Holland et al. reported that when an interscalene block was combined with either 4 mg or 8 mg of dexamethasone, there was no significant difference in the duration of anesthesia between the two groups [15]. In this study, the combined use of 3.3 mg of intravenous dexamethasone significantly extended the duration of anesthesia, and no obvious adverse events were observed.

There have been several reports evaluating complications due to concomitant use of dexamethasone as a secondary outcome [8,9,13,15,16]. Desmet et al. reported that when an interscalene block was combined with intravenous dexamethasone at doses of 1.25, 2.5, or 10 mg, blood glucose levels remained within the normal range for all doses [14]. On the other hand, patients with diabetes were excluded, and the effect of concomitant use of dexamethasone on blood glucose levels in patients with diabetes is unknown. This study also excluded cases with a relatively high risk of infection, such as those with diabetes or those undergoing reoperation, and further investigation is required to determine whether dexamethasone can be safely administered in such cases.

This study has several limitations. First, this is a retrospective study with a small number of cases. Second, the anesthetic agents used for the interscalene block were not standardized between the two groups, and the procedure was performed by multiple anesthesiologists. In this study, ropivacaine or levobupivacaine was used as the anesthetic agent, selected according to the preference of the anesthetist. Although there was no significant difference in the proportion of anesthetics selected between the two groups, there have been reports that levobupivacaine had a longer analgesic effect from nerve blocks than ropivacaine [17], and it cannot be denied that differences in anesthetics may have influenced the results of this study. Third, we were unable to assess changes in blood glucose levels.

## Conclusions

The group that also received intravenous dexamethasone had a significantly lower frequency of postoperative use of painkillers. The combination of interscalene block and intravenous dexamethasone injection is expected to extend the pain-relief duration of the block and may be an effective method for pain control after arthroscopic rotator cuff repair.
